# The Effect of Micro-Alloying and Surface Finishes on the Thermal Cycling Reliability of Doped SAC Solder Alloys

**DOI:** 10.3390/ma15196759

**Published:** 2022-09-29

**Authors:** Francy John Akkara, Sa’d Hamasha, Ali Alahmer, John Evans, Mohamed El Amine Belhadi, Xin Wei

**Affiliations:** 1 Department of Industrial and Systems Engineering, Auburn University, Auburn, AL 36849, USA; 2 Department of Mechanical Engineering, Faculty of Engineering, Tafila Technical University, Tafila 66110, Jordan

**Keywords:** surface finishes, micro-alloy, ENIG, microstructure, SAC, reliability

## Abstract

The surface finish (SF) becomes a part of the solder joint during assembly and improves the component’s reliability. Furthermore, the SF influences the solder joint’s reliability by affecting the thickness of the intermetallic compound (IMC) layer at the solder interface and copper pads. In this experiment, five different alloys are used and compared with the SAC305 alloy, two of which, Innolot and SAC-Bi, are bi-based solder alloys. This study includes three common SFs: electroless nickel immersion gold (ENIG), immersion silver (ImAg), and organic solderability preserve (OSP). The performance of three surface finishes is examined in terms of component characteristic life. All of the boards were isothermally aged for twelve months at 125 °C. The boards were then exposed to 5000 cycles of thermal cycling at temperatures ranging from −40–+125 °C. Most of the current research considers only one or two factors affecting the reliability of the electronic package. This study combines the effect of multiple factors, including solder paste content, SF, isothermal aging, and thermal cycling, to ensure that the test conditions represent real-world applications. In addition, the electronics packages are assembled using commercialized alloys. The current study focuses on a high-performance alloy already present in the electronic market. The failure data were analyzed statistically using the Weibull distribution and design of experiments (DOE) analysis of variance (ANOVA) techniques. The findings reveal that the micro and uniformly distributed precipitates in solder microstructures are critical for high-reliability solder joints. Re-crystallization of the thermally cycled solder joints promotes the local formation of numerous new grains in stress-concentrated zones. As the fracture spreads along these grain boundaries and eventually fails, these new grains participate in crack propagation. Aging significantly worsens this situation. Finally, although the ENIG surface finish with its Ni layer outperforms other SFs, this does not imply that ENIG is more reliable in all solder paste/sphere/finish combinations.

## 1. Introduction

The reliability of solder joints is a critical concern, particularly in harsh environments. The fundamental requirement of solder joints is to absorb the strains caused by thermal expansion mismatches between various materials [1]. Temperature changes can be caused by the environment or power cycling. The structural characteristics of the solder joint are influenced by thermal cycling. After thermal cycling, different failure modes, such as fatigue/creep processes and failure owing to shear stress, were detected in the microstructure [2]. Aging exacerbates this problem by affecting the mechanical and physical characteristics of the solder substance.

The coarsening of the precipitate and the formation of the brittle IMC layer weaken the solder joint over time, reducing its reliability. Furthermore, aging changes the structural characteristics of solder. Following the reflow process, copper from the pad dissolved into the bulk solder and developed an intermetallic compound (IMC) at the interface. The IMC layer ensures that the solder and the substrate have strong metallurgical bonding [3,4]. This IMC layer has continued to develop during the reflow operation, which was detrimental to the strength of the joint owing to its brittle nature. To make matters even worse, the formation of the IMC layer is accelerated by the process of aging [5,6]. To minimize the adverse effects of aging, several elements, including bismuth (Bi), nickel (Ni), antimony (Sb), cobalt (Co), and indium (In), have been micro-alloyed with the SnAgCu (SAC)-based solder alloy [7,8,9,10]. Among several materials, SAC-based solder alloys have shown positive prospects. Kariya et al. [11] investigated the impact of the silver (Ag) content on the shear fatigue characteristics. The results exhibited that SAC105 and SAC305 were the most commonly used solder alloys. As the amount of Ag in the alloy increases, its strength increased, making it more brittle. Yongping et al. [12] determined that 1 percent Ag is superior to 3 percent Ag for drop tests. However, Otiaba et al. [13] found that in the case of thermal cycling, fatigue resistance increased with the Ag concentration. The results showed that SAC305 is more effective for thermal cycling than SAC105. The drawback of this research is that because a solder joint also encompasses solder spheres and surface finishes in addition to solder paste, the impact of these parameters on reliability should also be considered. Akkara et al. [14] stated that the electroless nickel immersion gold (ENIG) surface finish was preferable to the others, which was attributable to the Ni coating that preventing copper diffusion. Akkara et al. [15] investigated the impact of combining solder spheres and surface finish (SF) with SAC-Bi pastes. The authors observed that solder spheres had a significantly more substantial influence on reliability than SF. However, this study did not address the impacts of aging. Sinan et al. [16] investigated the fatigue behavior of individual solder joints. The results showed that the fatigue resistance of the organic solderability preserve (OSP) and immersion silver (ImAg) surface finishes for the SAC-Bi solder joints surpassed the ENIG surface finish. However, the fatigue life of the SAC- Bi alloys was poor for the ENIG surface finish due to the brittle nature of the IMC-Ni interface. Francy et al. [14] studied the influence of the surface finish on thermal cycling. The findings demonstrated that the ENIG surface finishes outperformed other finishes in terms of fatigue life performance, independent of the SAC content. Zhang et al. [17] examined the effects of aging temperature on the reliability of solder joints. It was observed that creep rates were higher at elevated aging temperatures and that alloys with a lower silver content were more susceptible to aging than alloys with a higher silver concentration. Furthermore, the authors found that aging considerably reduced the fatigue life of solder joints owing to precipitate coarsening and IMC layer development. When the particles are fine and small, they efficiently limit dislocation motion, minimize grain sliding, and strengthen the material. However, as they become larger and coarser, their ability to block dislocation motions and grain boundary sliding decreases, lowering the strength and creep resistance of the material. Sinan et al. [6] reported that aging at room temperature had degradation effects. The study revealed that dopants reduced the effects of aging while also increasing the inelastic work per cycle and plastic strain range. Aging at higher temperatures exacerbates these negative impacts. Few studies have examined the consequences of recrystallization during thermal cycling. Mattila et al. [18] indicated that the new grain boundaries resulting from recrystallization offered ideal locations for fracture propagation with less energy consumption than crack propagation in the as-soldered microstructure. The copper traces were protected using solder masks. However, the copper pads remain unprotected. The SF was utilized to protect the copper pads from addressing this issue. In addition to protecting the copper pads from oxidation, the SF increases solderability by dissolving the surface finish’s outermost layer during soldering. Occasionally, SFs have considered solder masks over bare copper (SMOBC). To accurately estimate the cost, quality, and reliability of a printed circuit assembly, it is necessary to choose the proper printed circuit board (PCB) surface finish. Consider the strengths and weaknesses of each surface finish when matching it to specific requirements.

After revising the published papers, it can be concluded that adding Ag in solder alloys was found to provide enhanced mechanical and fatigue properties [11,16]. However, the drop shock test indicated the detrimental effect of Ag with the reduction in the ductility [12]. The other micro-alloying elements, such as Bi, Ni, and Sb, were also reported to improve the reliability of solder joints [2,6,16]. Meanwhile, these elements could prevent the degradation of properties during aging [6,14,15]. ENIG surface finishes outperformed OSP and ImAg surface finishes in the thermal cycling test [14], while the converse conclusion was drawn from the mechanical shear fatigue test [16]. The failure mechanism of solder joints in thermal cycling tests still needs a clear understanding. Table 1 compares the present study to those of other researchers described in the literature.

In this study, thermal cycling of the BGA components was performed after one year of aging. Factors influencing solder joint reliability, such as solder paste, solder sphere, SF, and aging, are considered to ensure that the test conditions represent real-world applications. The main objectives of this study could be described as follows: examine the impact of the SFs, including ENIG, ImAg, and OSP, on component reliability from the perspective of IMC growth. Furthermore, we investigated the impact of Bi content in the solder alloy composition on component reliability. To meet our study goals, the failure data collected were analyzed statistically, utilizing Weibull distribution and design of experiments (DOE) analysis of variance (ANOVA) techniques to investigate the impact of all parameters and their interactions on solder joint reliability. Following the test, the failed components were cross-sectioned and studied using optical and scanning electron microscopy to better understand the various failure modes.

## 2. Experimental Setup, Equipment, and Procedure

### 2.1. Test Samples and Preparation

**Figure 1:**
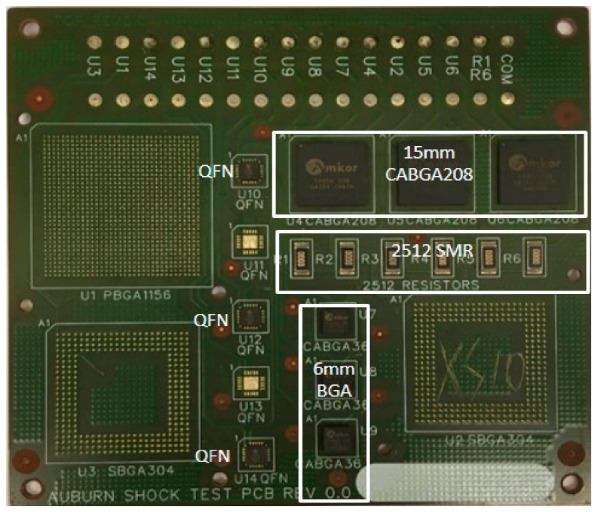
Components assembled on the PCB board.

The test vehicle’s PCB comprises four layers of FR4-06 glass-epoxy substrate with a glass transition temperature of 170 °C and dimensions of 10.16 cm × 12.7 cm × 0.16 cm. The test vehicle, as displayed in Figure 1 was outfitted with non-solder mask defined (NSMD) pads. Three different SFs, namely ENIG, ImAg, and OSP, were used. Rutherford backscattering spectroscopy (RBS) was used to determine the SF thickness. The RBS results show relatively sharp spectra for the SFs, but less sharp spectra for the Au and Ni RBS peaks. The less sharp RBS peaks could indicate interfacial roughness and/or a slight inter-layer diffusion. The copper (Cu) pad is coated with 3 µm of pure Ni, and 800 A° of pure Au for the ENIG finish. The copper in the case of ImAg was coated with 0.35 µm of Ag. The OSP finish was the least thick, including 600 A° of organic material. The 15 mm × 15 mm chip array ball grid array (CABGA208) with a 0.8 mm pitch was used in the study. All the components are daisy chained to guarantee that all solder joints are included in the circuit and to enable continuous sampling of component resistance to identify interconnection failure. Five solder alloys included in this study provide insight into the impacts of micro-alloying new elements with the SAC alloy to enhance reliability. Bi-based solder alloys were used in two of the alloys, which were compared to the SAC305 alloy. The compositions of the solder alloy and the test matrix are listed in Table 2. All of the boards were isothermally aged at 125 °C for twelve months. A temperature of 125 °C was selected in order for the packages to experience effective aging. Furthermore, in high-reliability applications where ten or more years of the product life is expected (e.g., automotive, military, and aerospace), the long-term reliability of lead-free solders is not well understood. In these applications, failures could be fatal and are never expected. Many studies have focused on aging at 125 °C at 3, 6, 12 and 24 months [19,20,21,22]. These studies compared the reliability performance and the degradation effect during isothermal aging. Subsequently, the boards were exposed to thermal cycling at temperatures ranging from −40–+125 °C for 5000 cycles.

### 2.2. Surface Mount Technology (SMT) Assembly

**Figure 2:**
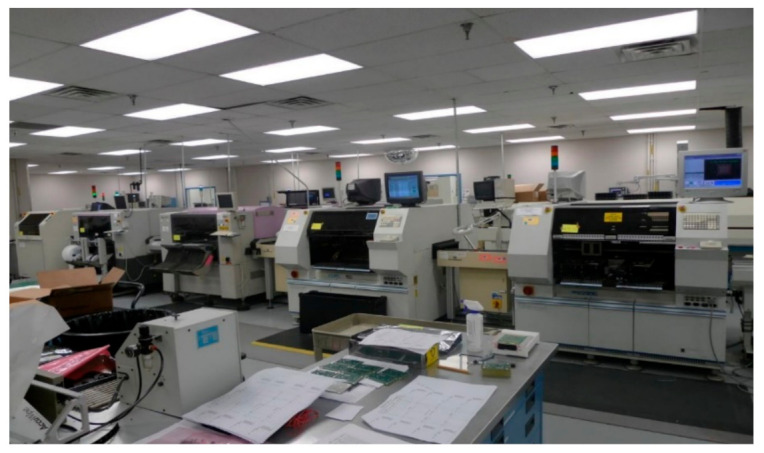
Assembly line at UAH.

**Figure 3:**
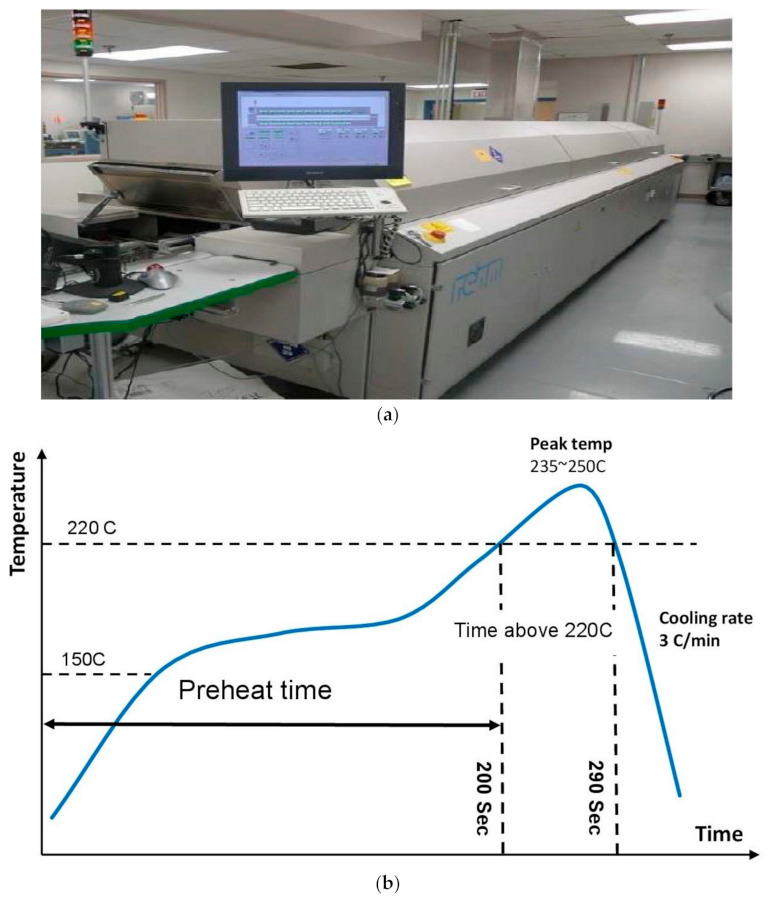
Schematic figure of (**a**) Reflow oven; and (**b**) Reflow profile.

The test boards were assembled at the University of Alabama in Huntsville’s (UAH) Electronics Packaging Lab. A 12-h ‘bake out’ operation at 150 °C was executed in the oven to eliminate moisture that could damage the assembly. A 6 mm-thick E-FAB Electroform stencil was used for stencil printing. The MPM UP2000 HiE (Precision Placement Machines, Inc., Fremont, NH, USA) was used to print the solder paste. The assembly was visually examined and validated using a 3D inspection procedure after the paste was printed on the PCB substrate to verify the area and volume of the paste deposit. All components were picked and placed using the Universal GSM-1 pick and place machine with a tray feeder. The machine uses a pre-programmed algorithm to precisely choose and place the components on the PCB board. The assembly was examined again to verify that there were no improper package placements. Figure 2 depicts the SMT assembly line at the UAH in Alabama. Following the placement of the components, the assembly was reflowed in a 13-zone Rehm V7 convection reflow oven with a conveyor speed of 26 inches/min that operated in a nitrogen atmosphere. The thermal reflow profile was chosen such that the solder joints exhibited the best wetting with the least amount of board damage. The reflow oven and the temperature profiles are shown in Figure 3. In general, the reflow profile consists of four steps. The pre-heating step lasted approximately 80 s, and the temperature was increased from 35–155 °C. This was followed by a 75-s soak stage ranging in temperatures from 155–175 °C. The maximum temperature is approximately 245 °C. The total duration over 217 °C, representing the melting point for SAC alloys, is approximately 70 s. The highest ramp rate is approximately 3 °C/s. The assembly was allowed to cool after the reflow. Afterward, the assembly is examined using transmission X-ray tomography to guarantee the integrity of the solder joints in the assemblies. This technique can identify common problems, such as inadequate solder, solder bridging, voids, dark pads, and head-in-pillow. In case of defects, the board was reworked.

### 2.3. Isothermal Aging and Thermal Cycling

**Figure 4:**
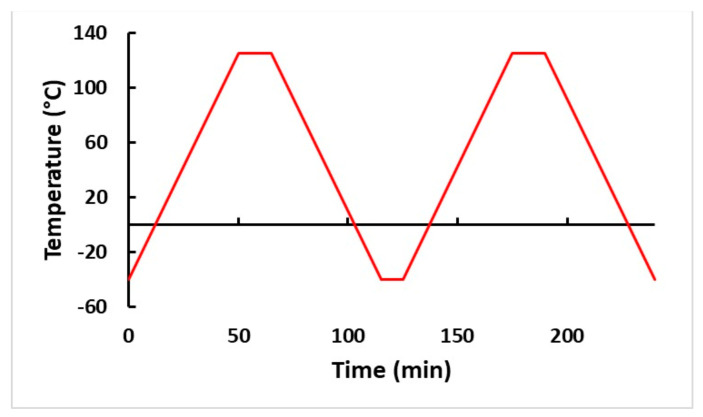
Thermal cycling profile.

Following assembly, the boards were isothermally aged for 12 months at 125 °C in a 16 ft^3^ Thermotron chamber. After aging, all boards were exposed to thermal cycling at temperatures ranging from −40–+125 °C, with 15 min. dwelling at +125 °C and 10 min dwelling at −40 °C. The ramp period for the profile was 50 min, at a rate of around 3.3 °C/min. The thermal profile was based on the modified JEDEC JESD 22-A104-B standard. Figure 4 depicts the thermal cycling profile of the test.

### 2.4. Data Acquisition System

Palettes were used to organize the test vehicles. All components were wired to the data acquisition system to monitor the component resistance continually. The system comprised a Keithley 2001 digital multimeter and a Keithley scanning system, which were connected through NI LabView software. The IPC-9701 standard defines a solder joint failure as an increase in resistance of more than 1000 ohms. This study described a solder joint failure as 1000 ohms increase in daisy chain resistance over the baseline resistance for five successive observations.

### 2.5. Microstructure Analysis

The failed samples were employed for microstructure analysis after the test. The failed piece was removed from the PCB using a saw from Allied High-Tech Products, Inc., (Compton, CA, USA) with a diamond sectioning blade. The sample was subsequently cleaned, and an optical microscope was used to select the face of the sample to be studied. The components were then mounted in an epoxy system (resin and hardener). The hardener and resin were mixed in a 1:6 ratio to prepare the epoxy. Each sample was labeled, and a Buehler sample holder clip was used to keep the sample upright. The epoxy was placed in a mold for 20–24 h for curing. After curing, the sample was removed from the mold and prepared for analysis. Semi-automated polishing equipment from Pace Technologies (Tucson, AZ, USA) (FEMTO-1000 with a NANO-1000T polishing head) and silicon carbide grit papers were utilized for grinding and polishing. Papers with grits of 120, 400, 600, 800, 1000, and 1200 were utilized progressively. After each grit paper, the sample was rotated by 90° such that the cuts caused by one paper were eliminated by the following finer grit paper.

After grinding, the sample was polished using Pace Technologies’ 3 µm, 1 µm, 0.05 µm viscous alumina polish, and finally with Buehler’s 0.02 µm colloidal silica polishing suspension. Each polishing step lasted between 15 and 30 min., depending on the amount of scratch removed from the previous stages. After the sample was prepared, it was analyzed using a ZEISS Axio Imager.M2m optical microscope (ZEISS Microscopy, Oberkochen, Germany) with an Axiocam 503 color microscope camera and ZENCore software (Allied High Tech Products, Inc., CA 90220, USA). Polarized images were also acquired to assess the recrystallization. Further microstructural analysis was performed using a Hitachi S-2460N scanning electron microscope (SEM). A carbon coating was applied to the samples before SEM examination to prevent charging in non-conductive materials. Carbon was coated using a DV-401Carbon coating system. Carbon tape was used to ensure that the sample was appropriately grounded. Carbon yarn, employed as a carbon source, is maintained around 48 mm from the sample’s surface to provide optimal coat thickness.

## 3. Results and Discussion

Three different methods were used to assess the impact of micro-alloying and surface finish on the thermal cycling reliability of doped SAC solder alloys: (i) Weibull analysis was used to quantify the performances of various solder alloy-surface finish combinations; (ii) an ANOVA analysis was also used to identify the factors that had the greatest impact on the component reliability; and (iii) SEM images of SAC-Bi under various surface finishes.

### 3.1. Weibull Analysis for Different Surface Finishes

The Weibull distribution is one of the most frequently utilized distributions when modeling failure data statistically. The probability density function of the Weibull distribution is defined as follows:

(1) $$f\left(t\right)=\frac{\beta }{\theta }{\left(\frac{t}{\theta }\right)}^{\beta -1}e^{-{\left(\frac{t}{\theta }\right)}^{\beta }}$$

where *β* is the Weibull shape parameter, and *θ* is the Weibull scale parameter. Weibull analysis was performed to quantify the performance of each combination of components, including solder paste, solder sphere, and surface finish. The Weibull analysis includes two parameters: the shape parameter (*β*) and the scale parameter (*θ*). The shape parameter determines the slope of the plot, and the scale parameter is the typical life, which can be defined as the time (cycles) at which 63.2% of samples failed. B10 life, or the period at which 10% of the population is expected to fail, is also determined for each combination to compare early failures.

**Figure 5:**
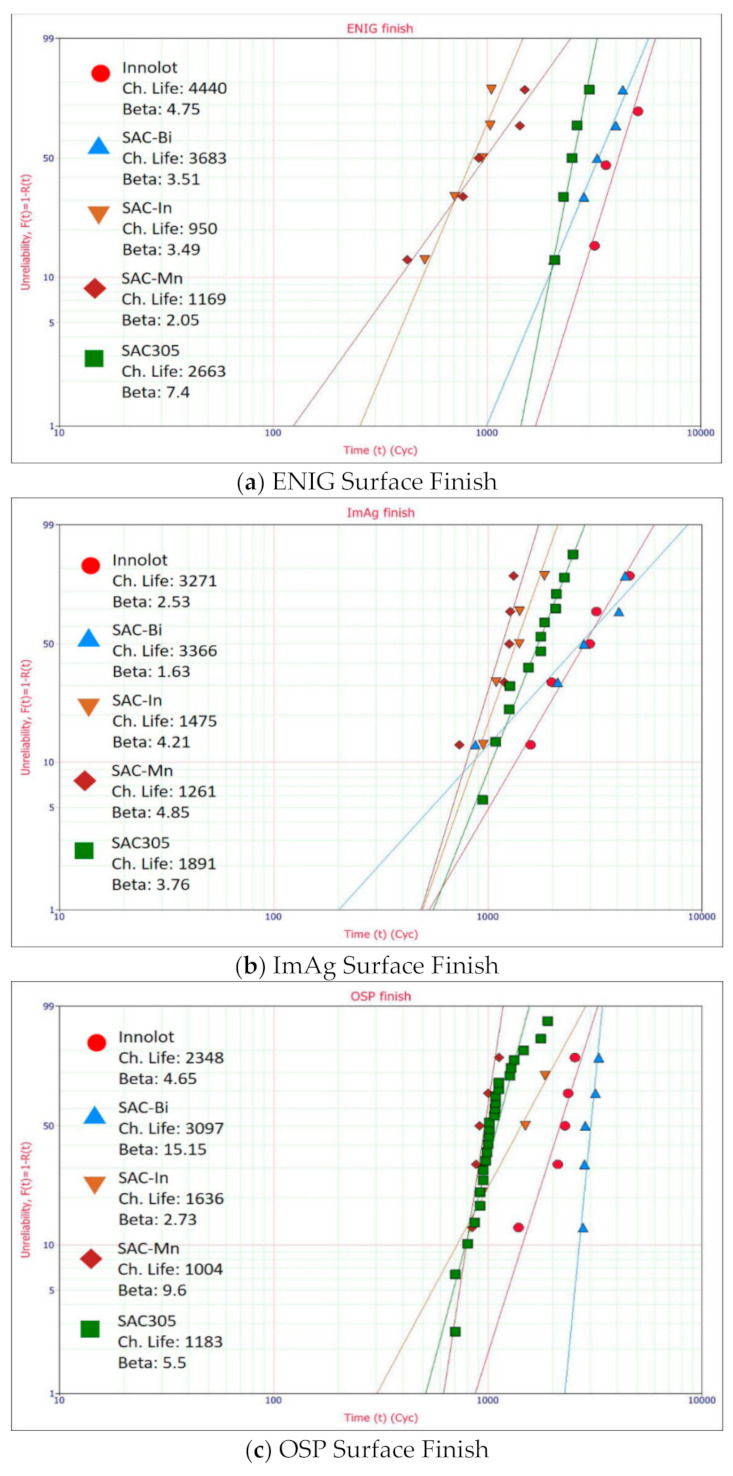
Weibull analysis for different alloys with; (**a**) ENIG; (**b**) ImAg; and (**c**) OSP surface finishes.

**Figure 6:**
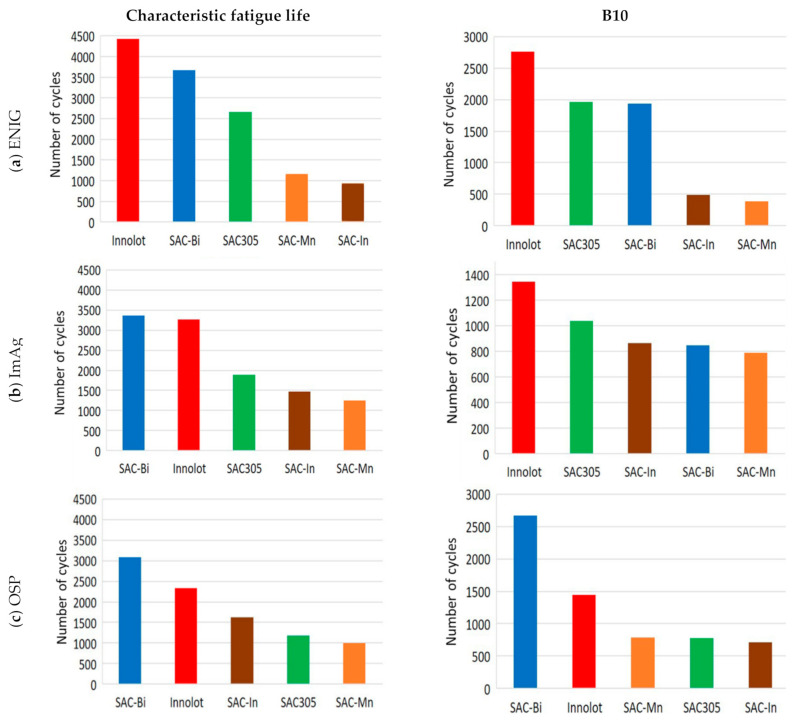
Summary of characteristic fatigue life and B10 for different alloys with, (**a**) ENIG; (**b**) ImAg, and (**c**) OSP surface finishes.

Figure 5a–c depict the Weibull analysis for several solder alloys under consideration for the ENIG, OSP, and ImAg surface finishes. With the same ENIG surface finish, SAC alloys containing Bi exhibited better thermal fatigue resistance than the others. A significantly greater characteristic fatigue life implies a superior performance. Innolot performed the best with the least failures, followed by SAC-Bi and SAC305. SAC-Mn and SAC-In were observed to provide the worst fatigue life but with similar performance. In the case of the ImAg surface finish, both SAC-Bi and Innolot demonstrated improved fatigue life, with SAC-Bi marginally exceeding Innolot. These alloys are followed by SAC305, and subsequently by non-Bi alloys. For the OSP surface finish, SAC-Bi once again outperformed. Innolot also showed a good fatigue life following SAC-Bi. However, SAC-In outperformed SAC305 in the opposite direction, which differs from the cases of ENIG and ImAg surface finishes. Figure 6 visualizes these findings more clearly. Furthermore, the B10 parameters were utilized to explain their early failures. Compared to SAC305, more early failures of SAC-Bi assemblies with ENIG surface finish were detected, resulting in reduced B10 life. In the case of the ImAg surface finish, an even worse B10 life of SAC-Bi was observed, which lies between those of the two non-Bi alloys. However, it is worth mentioning that SAC-Bi, with an OSP surface finish, provided the most robust connections. The addition of Bi to SAC solder alloys significantly enhanced both the B10 life and typical fatigue life, as demonstrated in Figure 6c. Furthermore, no significant variation in B10 fatigue life was observed across the three Bi-free solder alloys.

### 3.2. ANOVA Analysis for IMC Growth

**Figure 7:**
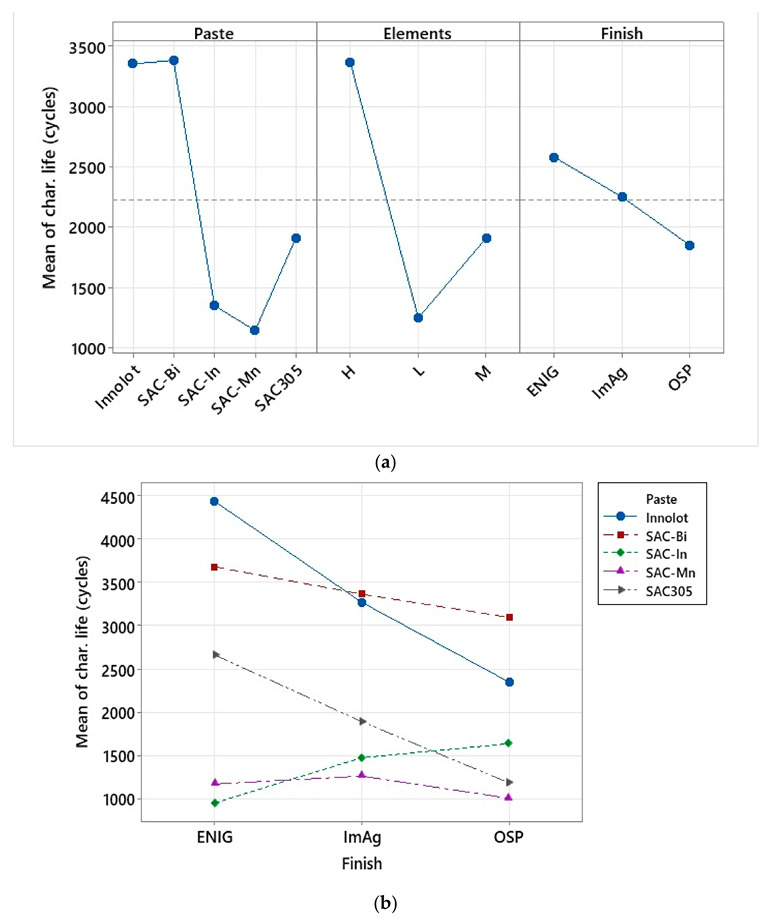
ANOVA analysis; (**a**) main effect plots including paste, elements, and surface finish; (**b**) Interaction effects plot for different alloys and surface finishes.

Figure 7a depicts the ANOVA analysis of the effect of different factors, such as solder paste, elements excluding Tin (Sn), and surface finish, on the characteristic life, while Figure 7b illustrates the interaction of various components. Table 3 shows how the elements were classified into three groups depending on their composition. Solder alloys with less than 4% elements were classified as low. Those between 4% and 7% were classified as medium. Finally, those with elements between 7% and 10% were classified as high. The main effects plot demonstrates that Innolot and SAC-Bi have superior thermal fatigue reliability due to the increased Ag and Bi contents in the two alloys. It has been found that when the Ag content increases, the β-Sn dendrites, and ternary microstructures become finer, offering improved reliability [23]. Coyle et al. [24] found that the dislocation pinning effect of Ag_3_Sn particles also escalates the characteristic thermal fatigue life. The inclusion of micro-alloying elements, such as Bi, Sb, and Ni, also improves the thermal fatigue resistance of solder joints owing to solid solution hardening and precipitation hardening in the β-Sn lattice [25,26]. In element does not seem to improve the reliability at the cost of a low Ag content. SAC-Mn performed the worst due to its low Ag content. Medium and low alloys exhibited degraded fatigue life in the absence of Bi, whereas high alloys demonstrated excellent performance with both high Ag and Bi contents. The ENIG surface finish performed best in terms of surface finish. The Ni layer prevents Cu diffusion from the Cu pads to the bulk solder. This inhibits the growth of the IMC layer. According to the interaction plot between the alloy and surface finish in Figure 7b, Innolot and SAC-Bi performed quite similarly in the case of the ENIG surface finish. In contrast, SAC-Bi performs slightly better than ImAg. SAC-Bi has a better life than Innolot in the case of the OSP surface finish. The combination of SAC alloys with Bi and SAC305 with ENIG seemed to perform the best. The pattern seems to be reversed for the other alloys, with OSP and ImAg outperforming the ENIG.

**Figure 8:**
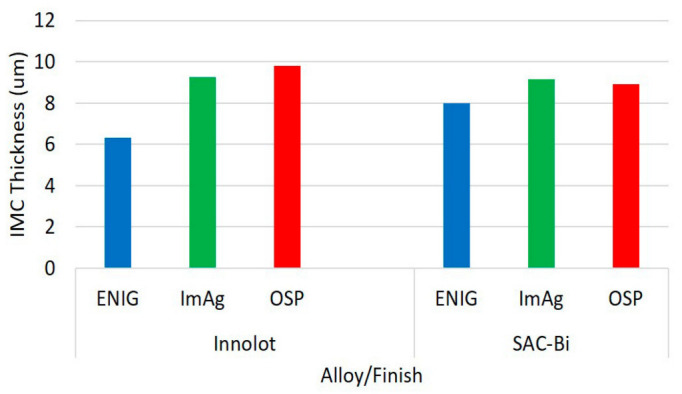
IMC layer thickness measurements.

**Figure 9:**
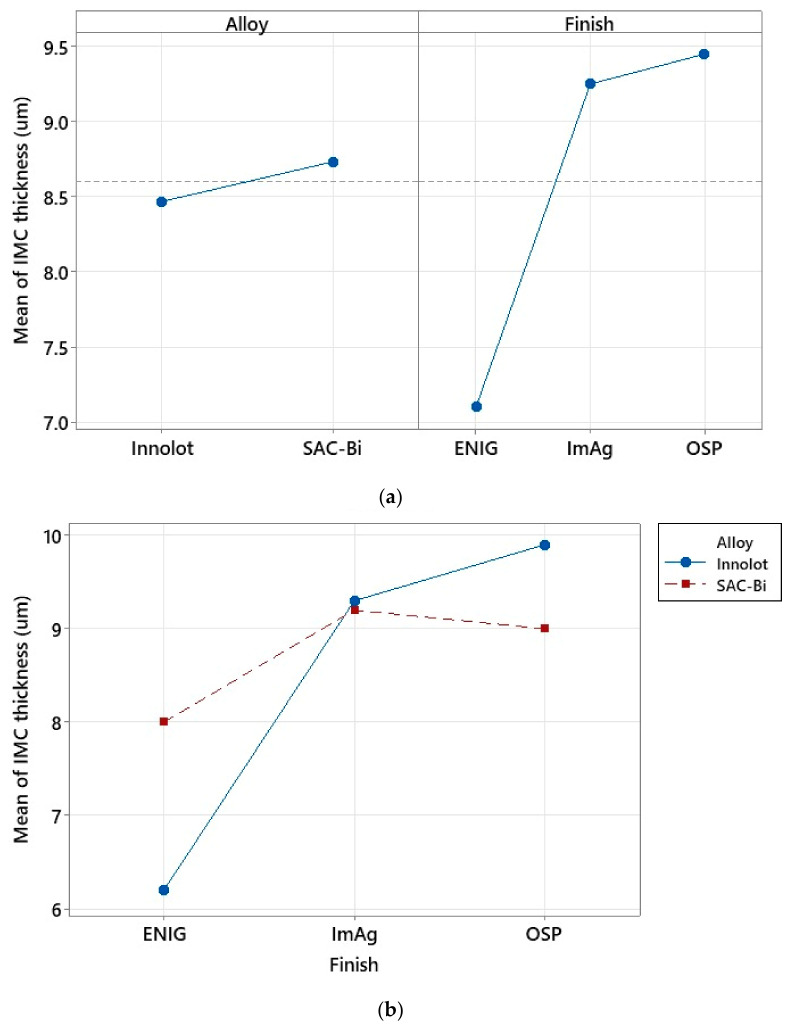
ANOVA analysis for IMC growth; (**a**) Main effects plot; and (**b**) Interaction plot.

Figure 8 exhibits the IMC layer thickness measurements of the Innolot and SAC-Bi solder joints for each surface finish. For each solder alloy, the ENIG finish had the thinnest IMC layer. The IMC thickness increased for the ImAg and OSP surface finish. In the case of the ENIG surface finish, the Ni layer seemed to have a considerable influence on the development of the IMC layer at the interface. Figure 9 examines the several elements that influence the growth of the IMC layer. It can be shown that the solder alloy or paste had no major effect on the IMC layer thickness in both cases within the same range. However, ENIG has the thinnest IMC layer, while ImAg and OSP have IMC layers that are more than 9 µm thick on average. This could be attributed to the Ni layer in the case of the ENIG surface finish. According to the interaction plot, SAC-Bi performs better with OSP than with Innolot regarding to having the lowest IMC thickness, and this tendency is reversed in the case of ENIG.

**Figure 10:**
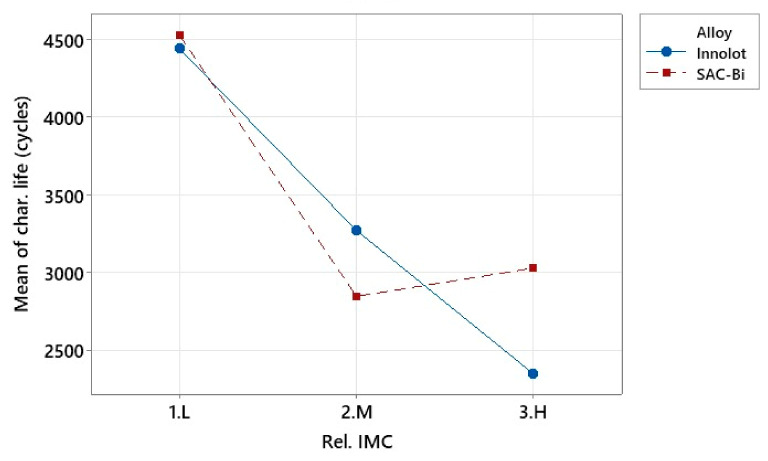
ANOVA analysis for interaction plot between alloy and rel. IMC layer thickness.

Figure 10 shows the ANOVA analysis for the effect of the relative IMC thickness and solder alloy on the characteristic life. It is shown that the thinnest IMC was associated with the highest typical life. The relative IMC thickness was classified locally for each alloy in this study. The thinnest IMC layer in each alloy was labeled low (L), while the thickest layer was labeled high (H). In the case of the thinnest IMC layer, the alloys performed similarly. Innolot outperformed SAC-Bi for the medium-thick layers. However, for the thickest IMC layer, SAC-Bi outperforms Innolot. The IMC layer was brittle. When the effect of Sb-containing Innolot is combined with the IMC layer, the solder joint becomes more brittle because Sb makes the alloy brittle [27].

### 3.3. Recrystallization and Microstructure Comparison

**Figure 11:**
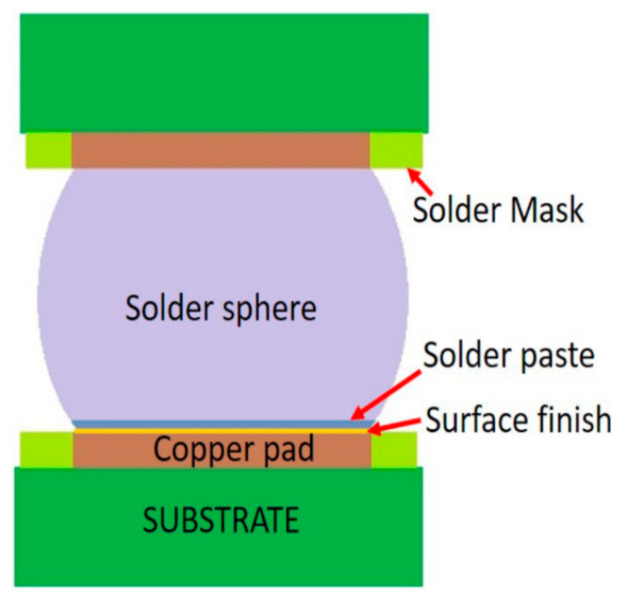
Typical solder joint.

Figure 11 depicts a typical solder joint. Several variables influence the reliability of the solder joints, such as the: solder sphere, solder paste, and surface finish. This study explored the influence of solder alloys (including solder spheres and pastes) under three surface finishes.

**Figure 12:**
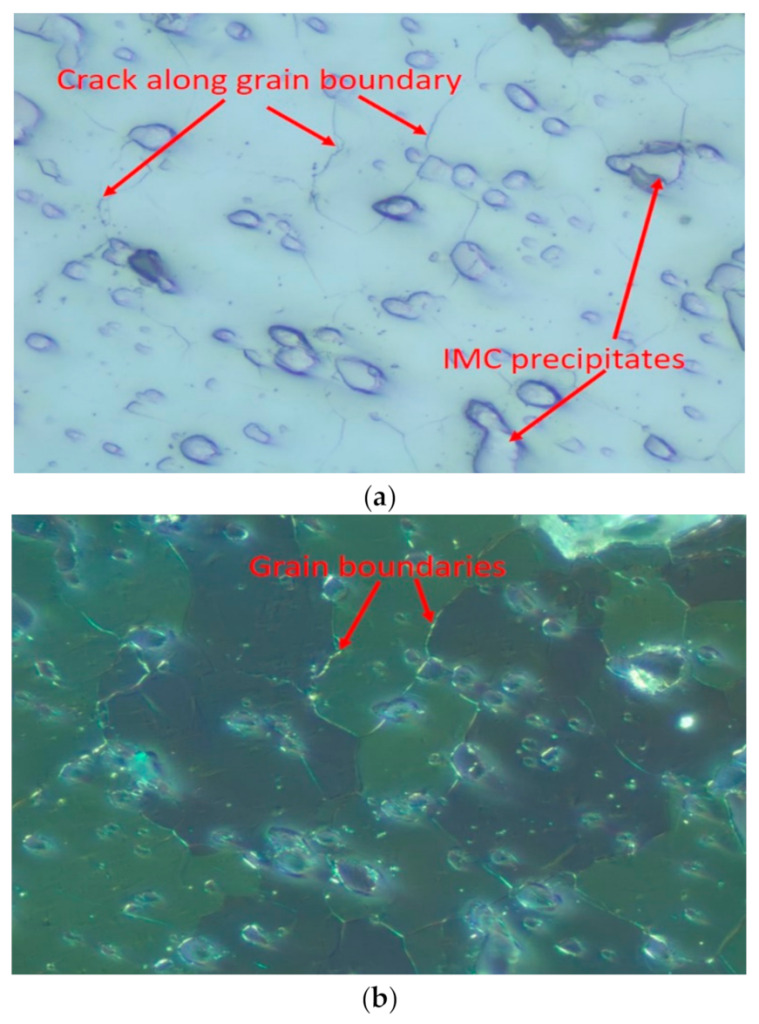
Schematic image of recrystallization after thermal cycling; (**a**) dark field image; and (**b**) polarized image.

Figure 12 portrays a typical solder alloy dark field and polarized image after thermal cycling. Thermally induced inelastic strain develops during thermal cycling due to material thermal expansion mismatches. Under such cyclic thermomechanical deformation, the β-Sn phase in the high-plastic deformation regions is more prone to dynamic recrystallization with more refined grains. These finer grains are more susceptible to creep deformation, such as grain boundary sliding or cracking. The recrystallized region provides an easy path for crack propagation. In this study, a crack initiated and propagated along the interface between the solder and the component, where there was an inelastic strain concentration [28].

**Figure 13:**
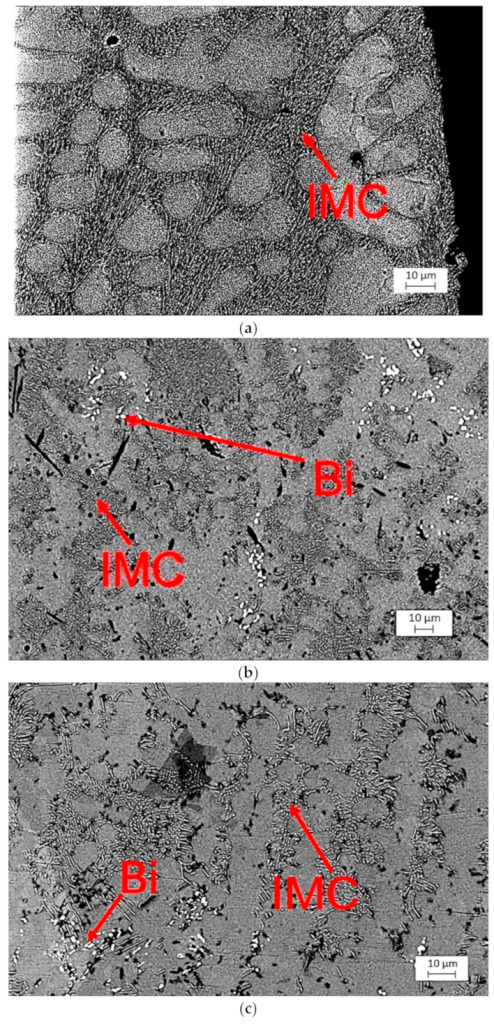
Microstructure comparison of several solder alloys. (**a**) SAC305; (**b**) SAC-Bi; (**c**) Innolot.

Figure 13 depicts the microstructures of the selected alloys after assembly. After the assembly, the precipitates were fine and evenly distributed. In the instance of SAC305, the IMC developed from an interlaced network. SAC305 showed a tertiary microstructure that included IMC precipitates, such as Cu_6_Sn_5_ and Ag_3_Sn, distributed in a β-Sn uniform dendritic structure [23]. SAC305 exhibited a precipitate hardening mechanism due to the presence of an Ag_3_Sn network in a β-Sn in bulk. Ag_3_Sn particles are the main microstructural features of SAC systems that influence thermal cycling aging [24]. This microstructure is ineffective for preventing dislocation movement [29]. The microstructures of SAC-Bi and Innolot appear to be similar to IMC precipitates forming a network due to the relatively high Ag content compared with that of SAC305 alloys. In addition, the bulk was characterized by the presence of Bi with Bi precipitates. These fine precipitates containing Bi were effective at preventing dislocation movements. It is worth noting that when the alloy aged, the fine IMC precipitates consolidated and coarsened.

### 3.4. IMC Morphology Characterization at Different Surface Finishes

**Figure 14:**
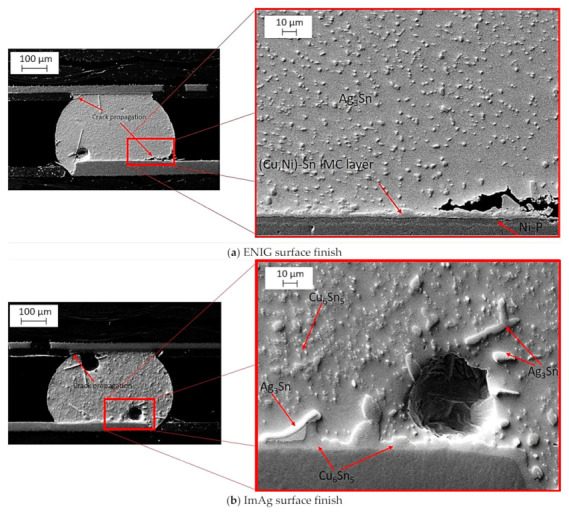

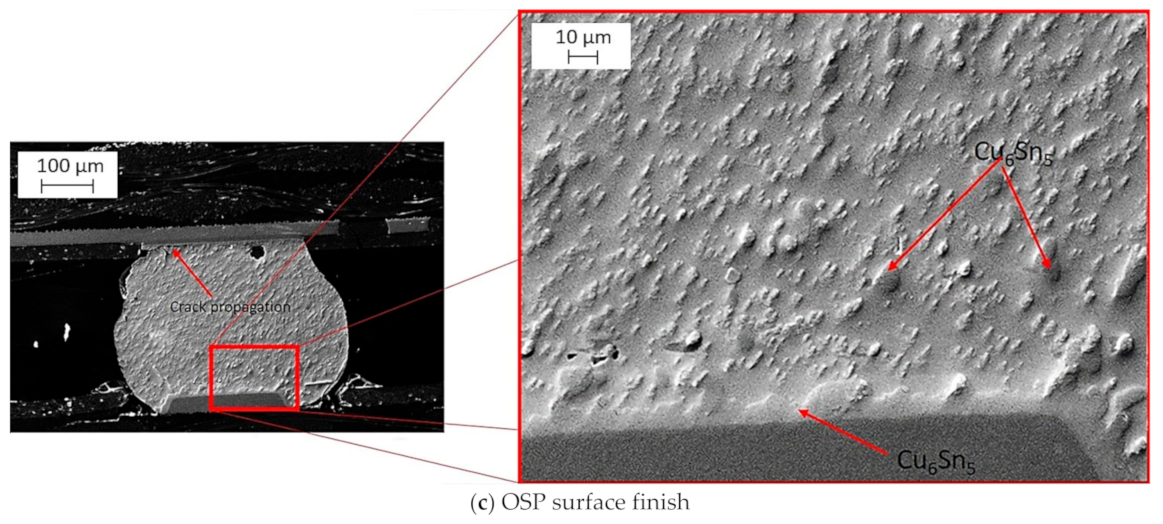
SEM image of SAC-Bi alloy with (**a**) ENIG; (**b**) ImAg; and (**c**) OSP surface finishes.

During the reflow process, an IMC layer was developed at the interface between the bulk solder and the board. This layer is required for electrical and mechanical interconnections. The IMC layer gradually thickens over time, and because of its brittle nature, it has a negative impact on component reliability. Figure 14 shows an SEM image of the SAC-Bi alloy after assembly with various surface finishes. As shown in Figure 14a, the ENIG surface finish contains a Ni layer that prevents Cu diffusion from the pads into the bulk solder to form Cu_6_Sn_5_ precipitates. Thus, the Cu in the IMC of the ENIG surface finish originated from the bulk solder rather than the Cu pads, and the IMC layer was composed of (Ni, Cu)_6_Sn_5_. In addition, the presence of a thin layer of gold protects the Ni during its shelf life. After the reflow, the gold was dissolved in the molten solder. The solid solubility of Au in Sn at ambient temperature is negligible; all of the Au precipitates as (Au, Ni) Sn_4_ [30]. Finer and uniformly distributed Ag_3_Sn precipitates were found in the bulk of the solder joint. In the case of the ImAg surface finish, as presented in Figure 14b, large precipitates of Ag_3_Sn were observed near the IMC layer due to the enhanced Ag content of the ImAg finish. Finally, as shown in Figure 14c, Cu from the pads diffused into the bulk to form an IMC layer at the pad-solder interface, as well as Cu_6_Sn_5_ precipitates in the bulk solder, similar to the ImAg surface finish, and a network of fine Ag_3_Sn particles was observed in the β tin matrix.

### 3.5. SEM Image of SAC-Bi under Different Surface Finishes

**Figure 15:**
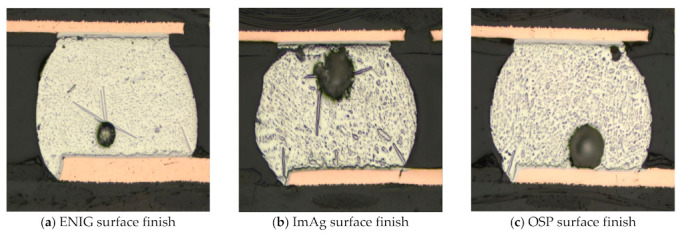
Comparison of SAC-Bi solder joints with different surface finishes with (**a**) ENIG; (**b**) ImAg; and (**c**) OSP.

Figure 15 displays darkfield images of solder joints at different surface finishes with ENIG, ImAg, and OSP. SAC-Bi alloys exhibited solid solution hardening appeared to be another hardening mechanism due to the presence of Bi in the bulk solder. The presence of Ag_3_Sn precipitates in bulk was found to be more uniformly distributed and finer in the case of ENIG or substantially coarser in the case of ImAg, and OSP finishes. This observation is consistent with the findings of Collins et al. [30]. The large number of needle-shaped Ag_3_Sn could also be observed in the ImAg surface finish after flow.

**Figure 16:**
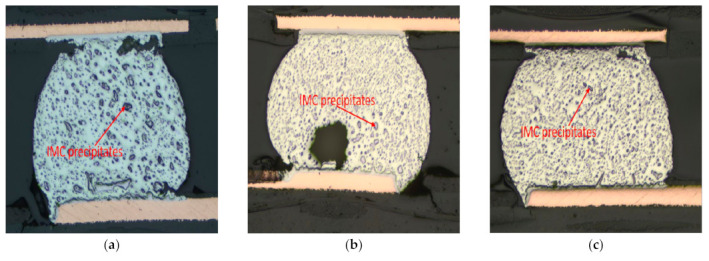
Cross-section images of different alloys; (**a**) SAC-In; (**b**) SAC-Bi; and (**c**) Innolot alloy.

Figure 16 depicts a cross-section of SAC-In, SAC-Bi, and Innolot alloy solder joints. Aging has caused the IMC precipitates to coarsen, and the images show that the coarsening differs according to the alloy. SAC-In showed coarsening of the IMC precipitate. The coarsening effect was on Cu_6_Sn_5_ substantially higher as compared to Ag_3_Sn. In addition, high recrystallization in the β Sn-rich region was noticed. Therefore, the formation of new grains provided an easy path for crack propagation, which caused a complete failure of the solder joints [23]. SAC-Bi and Innolot exhibited less coarsening of the IMC particles. Innolot demonstrated a formation of large teardrop-shaped Ag3Sn particles precipitates in the β Sn phase. Fine, evenly distributed precipitates are more effective at blocking dislocation movements, and the quantity of coarsening varies, as does the typical life of these alloys. The presence of Sb mitigated the coarsening effect due to its higher affinity for Sn. The formation of Sn-Sb compounds reduces the activity of Sn atoms and decreases the overall coarsening of Ag_3_Sn intermetallic [31].

**Figure 17:**
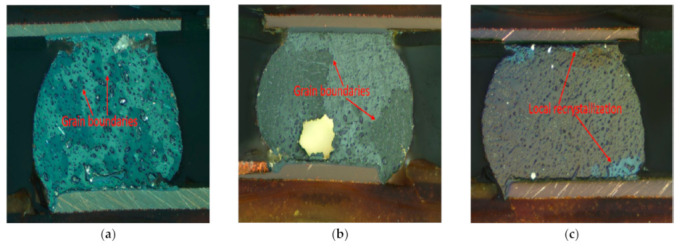
Polarized cross-section images of different alloys; (**a**) SAC-In; (**b**) SAC-Bi; and (**c**) Innolot alloy.

Figure 17 shows the polarized images of the SAC-In, SACBi, and Innolot alloys. Because of the thermally induced strain, numerous sub-grains develop during thermal cycling [32]. In the case of solder joints, these strains are concentrated along the solder/component interface, which contains NSMD pads. Gradually, a fracture is developed in this zone, propagating along the grain boundaries and resulting in failure. The composition and precipitates have a significant effect on preventing grain slippage and fracture development. The polarized images show that the quantity of recrystallization depends on the alloy composition. SAC-In seems to have the maximum recrystallization, followed by SAC-Bi, while Innolot tends to have the least amount of recrystallization. Terashima et al. [32] investigated recrystallization in thermal fatigue in Sn-1.2Ag-0.5Cu-0.05Ni and Sn-1.2Ag-0.5Cu solders, concluded that the increase in thermal fatigue life due to Ni addition resulted in not from fine Sn grain formation before thermal fatigue, but from the suppression of Sn grain growth after recrystallization. Furthermore, the authors observed that adding 0.05 mass % Ni decreased the coarsening and recrystallization in SAC-based alloys. These findings are consistent with the study finding that the Ni content of Innolot contributed to reducing recrystallization. Furthermore, microelements such as Bi, Sb, and Ni provide solid strengthening to compensate for the strength loss following the coarsening of Ag_3_Sn [24]. This disparity in microstructure is reflected in the fatigue life.

## 4. Conclusion

The influence of the surface finish (ENIG, ImAg, and OSP) and Bi content in the solder paste on the component reliability was thoroughly explored. A thermal cycling test was also performed to investigate the component reliability. The following conclusions could be drawn:

- The surface finish has a substantial influence on the reliability of components. The ENIG surface finish was the most reliable, followed by that of ImAg and OSP.
- The ENIG surface finish was associated with the least thick IMC layer due to the additional Ni layer. For Innolot solder, the OSP surface finish was 40% thicker than the ENIG surface finish. However, SAC-Bi alloys ENIG surface finish was 10% thinner than the OSP surface finish.
- Innolot, including Bi, Sb, and Ni, with ENIG surface finish, exhibited the highest thermal cycling reliability with a characteristic life of 4440 cycles, followed by SAC-Bi with a fatigue life of 3683 cycles. Accordingly, SAC-Bi had more early failures than Innolot.
- SAC-Mn and SAC-In behaved better with OSP than with ENIG compared to the other tested alloys.
- The joints with ENIG had a finer microstructure, as the finish prevented the diffusion of Cu from the pads due to the presence of Ni barrier between the Cu pad and the IMC layer.
- Higher reliability was associated with alloys with more micro-alloyed elements (Bi, Sb, Ni) due to the strengthening and hardening effect of Bi and the Sb in the solid solution.
- Crack propagation occurred along the grain boundaries formed by re-crystallization in regions of high plastic deformation due to accumulated strains at the interface and repeated exposure to elevated temperatures during accelerated temperature cycling (ATC).

## Figures and Tables

**Table 1:** Comparison of current study with other researchers as described in the literature.

Reference	Solder Alloy	Solder Paste	Surface Finish	Test Method	Remarks
Su et al. [6]	Sn-1.0Ag-0.5Cu, Sn-3.0Ag-0.5Cu, Sn-1.2Ag-0.5Cu-0.05Ni, Sn-0.3Ag-0.7Cu-0.05Ni-0.08Bi Sn-3.6Ag-0.74Cu-2.83Bi-1.48Sb-0.1Ni	N/A	OSP	Aged at 25 °C for 4 years, Shear fatigue	Aging leads to increased inelastic work per cycle and plastic strain range, thus less fatigue life. Alloys with more micro-alloying elements show the least life degradation.
Kariya et al. [11]	Sn-1.0Ag-0.5Cu, Sn-2.0Ag-0.5Cu, Sn-3.0Ag-0.5Cu, Sn-4.0Ag-0.5Cu	N/A	Cr/Ni/Au on aluminum elec- trodes for chip side and Ni/Au for copper traces on the substrate.	Non-aged, Shear fatigue	As the amount of Ag in the alloy increases, its strength increases, making it more brittle.
Yongping et al. [12]	Sn-1.0Ag-0.5Cu, Sn-3.0Ag-0.5Cu, Sn-0.3Ag-0.7Cu,	N/A	N/A	Non-aged, Drop test	The increased Ag content reduces failure resistance under drop conditions and a thicker IMC layer.
Otiaba et al. [13]	Sn-3.0Ag-0.5Cu, Sn-4.0Ag-0.5Cu	N/A	N/A	Non-aged, FEA thermal cycling simulation	SAC305 experienced a larger accumulated plastic work per cycle than SAC405, thus less thermal fatigue resistance.
Akkara et al. [14]	Sn-1.0Ag-0.5Cu, Sn-3.0Ag-0.5Cu, Sn-3.8Ag-0.7Cu-3.0Bi-1.4Sb-0.15Ni, Sn-3.8Ag-0.8Cu-3.0Bi Sn-2.5Ag-0.5Cu-2.0In-0.03Nd	3.0Bi-1.4Sb-0.15Ni, Sn-3.8Ag-0.8Cu-3.0Bi Sn-2.5Ag-0.5Cu-2.0In-0.03Nd	OSP, ImAg, ENIG	Aged at 125°C for 12 months, Thermal cycling	The addition of Bi improves fatigue resistance and slows down the adverse effect of aging and thermal cycling. The ENIG surface finish outperformed the OSP and ImAg surface finishes in most cases.
Akkara et al. [15]	Sn-1.0Ag-0.5Cu, Sn-3.0Ag-0.5Cu, Sn-3.8Ag-0.7Cu-3.0Bi-1.4Sb-0.15Ni, Sn-3.8Ag-0.8Cu-3.0Bi Sn-3.4Ag-0.5Cu-3.3Bi Sn-3.0Ag-3.0Bi-0.8Cu-Ni	Sn-3.8Ag-0.7Cu-3.0Bi-1.4Sb-0.15Ni, Sn-3.8Ag-0.8Cu-3.0Bi Sn-3.4Ag-0.5Cu-3.3Bi Sn-3.0Ag-3.0Bi-0.8Cu-Ni	ImAg, OSP	Aged at 125°C for 12 months, Thermal cycling	Recrystallization and precipitate formation lead to failures. Solder spheres showed more impact on the reliability than surface finish.
Su et al. [16]	Sn-3.0Ag-0.5Cu, Sn-3.5Ag-0.7Cu-3.0Bi-1.5Sb-0.125Ni, Sn-3.41Ag-0.52Cu-3.3Bi Sn-0.92Cu-2.46Bi Sn-0.3Ag-0.7Cu-0.05Ni-0.08Bi	N/A	OSP, ImAg, ENIG	Non-aged, Shear fatigue	The fatigue resistance of the solder joints with OSP and ImAg surface finishes outperformed ENIG surface finish. Solder alloys with higher Ag and Bi content demonstrate better fatigue life.
Zhang et al. [17]	Sn-37Pb Sn-1.0Ag-0.5Cu, Sn-2.0Ag-0.5Cu, Sn-3.0Ag-0.5Cu, Sn-4.0Ag-0.5Cu	N/A	N/A	Aged at 25°C, 75°C, 100°C, and 125°C for a period time of 0, 1, 2, 3, and 4 months, Creep test	SAC alloys with lower Ag content are more sensitive to aging than SAC alloys with higher Ag content. Lowering the Ag content of a SAC alloy causes higher creep rates for all aging conditions.
Mattila et al. [18]	Sn-3.0Ag-0.5Cu	Sn-3.8Ag-0.5Cu	OSP	Non-aged, Thermal cycling	Recrystallization creates new grain structures, providing an easy path for cracking propagation with less energy consumption.
Current Study	Sn-3.0Ag-0.5Cu, Sn-3.80Ag-0.70Cu-0.15Ni-1.40Sb-3.00Bi, Sn-3.41Ag-0.52Cu-3.3Bi, Sn-2.5Ag-0.5Cu-2ln-0.03Nd, Sn-0.5Ag-1.0Cu-0.03Mn	Sn-3.0Ag-0.5Cu, Sn-3.80Ag-0.70Cu-0.15Ni-1.40Sb-3.00Bi, Sn-3.41Ag-0.52Cu-3.3Bi, Sn-2.5Ag-0.5Cu-2ln-0.03Nd, Sn-0.5Ag-1.0Cu-0.03Mn	ENIG, ImAg, and OSP	Aged at 125°C for 12 months, Thermal cycling	ENIG surface finish was the most reliable, followed by ImAg and OSP. Innolot, including Bi, Sb, and Ni, had the highest thermal cycling reliability, followed by SAC-Bi.

**Table 2:** Composition and testing matrix of solder alloys.

Component	Solder Paste	Label	Composition	Surface Finish
CABGA208	A_Inn	Innolot	Sn-3.80Ag-0.70Cu-0.15Ni-1.40Sb-3.00Bi	ENIG ImAg OSP
	Ac_Cyx	SAC-Bi	Sn-3.41Ag-0.52Cu-3.3Bi	
	Hs_HT	SAC-ln	Sn-2.5Ag-0.5Cu-2ln-0.03Nd	
	Ind_1	SAC-Mn	Sn-0.5Ag-1.0Cu-0.03Mn	
	SAC305	SAC305	Sn-3.0Ag-0.5Cu	

**Table 3:** Element content.

Alloy	(100-Sn) %	High/Med/Low
Innolot	9.05	H
SAC-Bi	7.2	H
SAC-ln	5.03	M
SAC-Mn	1.53	L
SAC305	3.5	L

## Data Availability

The authors confirm that the data supporting the findings of this study are available within the article or upon request from the corresponding author.

## Nomenclatures

Ag	Silver
ANOVA	Analysis of variance
ATC	Accelerated temperature cycling
BGA	Ball grid array
Bi	Bismuth
CABGA	Chip array ball grid array
Co	Cobalt
Cu	Copper
DOE	Design of experiment
ENIG	Electroless nickel-immersion silver
ImAg	Immersion silver
Fe	Iron
I/O	Input/output
IMC	Intermetallic compound
In	indium
Ni	Nickel
NSMD	Non-solder mask defined
OSP	Organic solderability preservative
Pb	Lead
PCB	Printed circuit board
RBS	Backscattering spectroscopy
SAC	SnAgCu
Sb	Antimony
SEM	Scanning electron microscopy
SF	Surface finish
SMOBC	Solder mask over bare copper
SMT	Surface mount technology
Sn	Tin
UAH	University of Alabama in Huntsville
β	Shape parameter in Weibull distribution
θ	Scale parameter in Weibull distribution
